# Phylogenetic patterns of emergence of new genes support a model of frequent *de novo* evolution

**DOI:** 10.1186/1471-2164-14-117

**Published:** 2013-02-21

**Authors:** Rafik Neme, Diethard Tautz

**Affiliations:** 1 Max-Planck Institute for Evolutionary Biology, August-Thienemannstrasse 2, Plön, 24306, Germany

## Abstract

**Background:**

New gene emergence is so far assumed to be mostly driven by duplication and divergence of existing genes. The possibility that entirely new genes could emerge out of the non-coding genomic background was long thought to be almost negligible. With the increasing availability of fully sequenced genomes across broad scales of phylogeny, it has become possible to systematically study the origin of new genes over time and thus revisit this question.

**Results:**

We have used phylostratigraphy to assess trends of gene evolution across successive phylogenetic phases, using mostly the well-annotated mouse genome as a reference. We find several significant general trends and confirm them for three other vertebrate genomes (humans, zebrafish and stickleback). Younger genes are shorter, both with respect to gene length, as well as to open reading frame length. They contain also fewer exons and have fewer recognizable domains. Average exon length, on the other hand, does not change much over time. Only the most recently evolved genes have longer exons and they are often associated with active promotor regions, i.e. are part of bidirectional promotors. We have also revisited the possibility that *de novo* evolution of genes could occur even within existing genes, by making use of an alternative reading frame (overprinting). We find several cases among the annotated Ensembl ORFs, where the new reading frame has emerged at a higher phylostratigraphic level than the original one. We discuss some of these overprinted genes, which include also the Hoxa9 gene where an alternative reading frame covering the homeobox has emerged within the lineage leading to rodents and primates (Euarchontoglires).

**Conclusions:**

We suggest that the overall trends of gene emergence are more compatible with a *de novo* evolution model for orphan genes than a general duplication-divergence model. Hence *de novo* evolution of genes appears to have occurred continuously throughout evolutionary time and should therefore be considered as a general mechanism for the emergence of new gene functions.

## Background

The hallmark of the signature of a new gene (or orphan gene) is that it arises at some time within the evolutionary lineage towards an extant organism and has no similarity with genes in organisms that have split before this time [1-3]. This distinguishes orphan genes from genes that arise through full or partial duplication processes to form paralogous genes or gene families [4,5]. It has been proposed that orphan genes are likely to play a major role in lineage specific adaptations [1-3,6] and thus contribute to evolutionary innovations. There are two major models of how orphan genes can arise [3]. The first is the duplication-divergence model, which assumes that they emerge through an initial duplication of other genes, but this is followed by rapid divergence, such that all similarity to the parent gene is lost [1]. The alternative is the *de novo* evolution model, which assumes that genes can directly arise out of non-coding DNA [7]. Although this second possibility seemed initially rather unlikely, such genes have been found in *Drosophila*[8-10], yeast [11,12], mouse [13], Plasmodium [14] plants [15] and humans [16-18]. In fact, there is now increasing evidence that *de novo* evolution may be rather frequent. Studies in yeast have suggested that a large number of transcripts without annotation are actively transcribed and translated [19,20] and that such transcripts could be a source for *de novo* gene emergence (called “proto-genes”) [7,20].

We have developed phylostratigraphy as a method that identifies the genes that have arisen at each stage of a series of phylogenetically relevant splitting events [21]. This allows to systematically study the characteristics of such genes over time [22-25]. Using this approach we found that gene emergence rates are particularly high in the youngest lineages, implying a very active process of *de novo* evolution, since the times considered for these youngest lineages are too short for the duplication-divergence model to apply [3]. This is in agreement with the proto-gene concept, where non-coding transcripts are considered as possible sources of new genes [19,20]. However, a study of emergence trends across the whole phylogeny is still missing.

In the present paper we use the mouse as a focal species, which has a particularly well annotated genome. We show that it is indeed possible to derive distinctive patterns for gene emergence, which appear to be generally in accordance with a *de novo* evolution model. As a special case of *de novo* evolution, we revisit the possibility that existing genes have developed an independent second reading frame. Evolution of new genes within such double reading frame arrangements have been known since some time [26,27] (called “overprinting” by [27]). They have been well studied in viruses [28,29], but several examples are also known from eukaryotes and have been studied in detail for some genes [30-32]. Chung et al. [33] provided a first systematic approach to identify such alternative reading frames (ARFs) in mammals and suggested 40 candidate genes which appeared to use ARFs. We find here that it is indeed possible to retrieve even among annotated genes additional cases of overprinting, where the alternative reading frame maps to a different phylostratum than the original reading frame. This suggests that existing genes may readily become templates for *de novo* evolution of new gene functions within them, further supporting the notion that *de novo* evolution of gene functions are possible.

## Results

The duplication-divergence versus the *de novo* evolution model for orphan gene emergence make some different predictions with respect to gene emergence over time, for example on length distributions and exon distributions, as detailed below. Apart of looking for such differential predictions, it is also of interest to assess general patterns, such as orphan gene distribution across the genome, as well as the emergence of associated promotors. Below, we describe first how we assign the genes to different age classes and then use this assignment to study gene emergence trends and patterns.

### Phylostratigraphy of mouse genes

**Figure 1:**
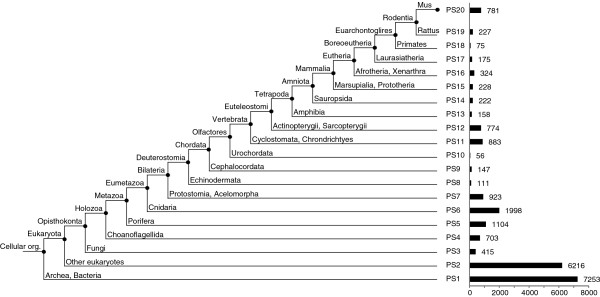
**Phylostratigraphy of the mouse genome.** Each phylostratum corresponds to a node in the phylogenetic tree of the species. Representative outgroups are named under each node. The bar graph to the right represents the number of annotated protein-coding genes mapped to the respective phylostratum at a blastp threshold of e < 10^-3^.

The phylostratigraphic approach was used to estimate the time of emergence of each of 20,775 annotated protein coding loci in the mouse genome (Figure 1). Twenty phylogenetic classes or phylostrata were defined according to consensus phylogenetic relationships between groups with enough available protein sequence information. The first phylostratum (ps1) represents the basis of all cellular life, i.e. the oldest genes, while the last phylostratum (ps20) represents the lineage leading to mouse since the split from rat blastp was used to assign for each mouse gene its presumptive origin within this phylostratigraphy. For this we use an e-value cutoff of < 10^-3^, which has previously been found to provide an optimal compromise between sensitivity and accuracy [1,34]. The results of the assignment to the respective phylostrata are listed in Additional file 1: Table S1 and summarized in Figure 1.

Approximately 60% of the annotated protein coding genes in the mouse genome originate from prokaryotic and basal eukaryotic ancestors (ps1-2). The rest of the genes have emerged later in the phylogenetic history, with peaks correlating to large scale biological transitions. For example, the peak around ps6 represents the single-cell to multicellular organism transition [23] and the peak around ps11-12 represents the invertebrate to vertebrate transition. Another peak is evident at ps20, representing all genes that have evolved since the rat/mouse split. Although this may partly be ascribed to annotation problems within the youngest group of genes [7] many of them are likely to represent *de novo* evolved genes, since mouse and rat are so close to each other that any duplicated gene would easily be traceable, even if it would evolve with the rate of a non-functional pseudogene.

### Genomic features across ages

**Figure 2:**
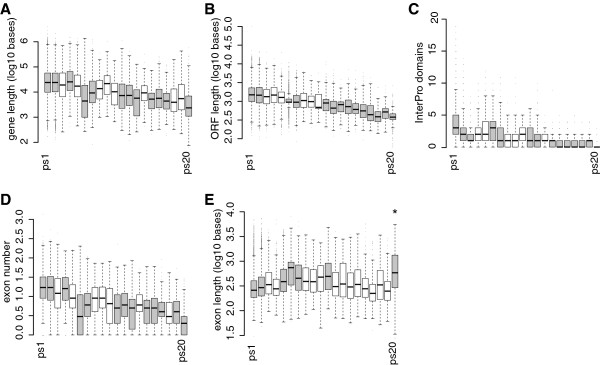
**Features of genes for different phylogenetic age groups in the mouse. A**. Gene length distributions (includes exons and introns); **B**. ORF length distributions; **C**. Median number of InterPro domains per gene; **D**. Median exon numbers per gene; **E**. Median exon lengths per gene. Box-whisker plots around median values (bars) with quartile ranges and outliers as dots. Significant (p < 0.01) distribution differences were found for ps20 (marked with *) in E (t-test). Gray bars indicate phylostrata with non-randomly distributed values for each variable, based on permutations (n = 10,000) and Kolmogorov-Smirnov tests (p<0.01).

We used the phylostratigraphic assignment of the genes to assess the emergence trends over time for several relevant gene features (Figure 2). Some of the gene features were selected to allow to distinguish the duplication-divergence model from the *de novo* model.

With respect to gene length, the *de novo* model would predict that younger genes should be shorter than older genes, since it is unlikely that complex protein sequences emerge *de novo*. Rather one would expect that they could increase in size over evolutionary time. In the duplication-divergence model one would not expect length-dependence over time, since long and short genes should be equally likely subject to duplication at any time level. The results show, however, a strong length-dependence over time, both with respect to gene length (Figure 2A) as well as open reading frame length (Figure 2B). The Spearman rank correlations across the 20 phylostrata are very high (Table 1) suggesting an almost continuous trend over time. Such trends for gene length distributions had also previously been noted in analyses using fewer age classes [35,36].

**Table 1:** List of overprinted genes detected via a phylostratigraphic approach based on annotated ORFs in Ensembl

**ENSMUS IDs**				**Phylostratum**		**Comment**
**Gene**	**Newer protein**	**Older protein**	**Gene name**	**Overprint**	**Original**	
G00000029642	P00000106186	P00000058355	Polr1d	5	2	Same start as main gene, but acquired additional exons
G00000030970	P00000127123	P00000033269	Ctbp2	12	1	Same start as main gene, but acquired an additional internal exon
G00000035504	P00000100994	P00000100995	Reep6	17	2	New initiation codon creates second reading frame
G00000089756	P00000104646	P00000104577	Gm8898	18	2	Same start, but new splice variant; paralog of Gm4723
G00000078898	P00000104676	P00000104675	Gm4723	18	2	Same start, but new splice variant; paralog of Gm8898
G00000038227	P00000133896	P00000046939	Hoxa9	18	2	New starting exon initiates a separate reading frame
G00000067786	P00000134415	P00000085836	Nnat	18	16	Same start, alternative splicing leads to new reading frames
G00000044405	P00000105110	P00000051732	Adig	20	16	Same start as main gene, but acquired an additional internal exon
G00000025144	P00000101761	P00000026137	Stra13	20	2	Gain of alternative second exon induces a shift from the older frame
G00000033720	P00000109417	P00000041872	Sfxn5	20	2	Alternative first exon and last exons, common second exon
G00000063235	P00000107087	P00000077036	Ptpmt1	20	1	Alternative transcription start site and start codon
G00000044303	P00000030237	P00000061847	Cdkn2a^a^	16	1	New starting exon initiates a separate reading frame. Also known as Arf, Pctr1, MTS1, Ink4a
G00000027523	P00000104716	P00000085184	Gnas^b^	18	2	New initiation codon creates second reading frame. Also known as Nesp, GPSA

^a^has previously been described, see [32,33].

^b^has previously been described, see [30,31,33].

A differential prediction can also be made for the expected correlation with protein domain emergence. *De novo* evolved proteins will initially have no domains which are shared with other genes, while duplicated genes would tend to retain domains of their parental genes [37]. Hence, the *de novo* evolution would predict domain gain over time, while no distinct pattern is expected for the duplication-divergence model. Again we find indeed a strong time-dependence with a continuous trend for domain emergence (Figure 2C; Table 1), supporting the *de novo* model.

*De novo* emerged genes should also have initially fewer exons, but could be expected to accumulate additional ones over time. In the duplication-divergence model, on the other hand, one would not expect a time dependency of exon numbers, since this mechanism should work the same at every time horizon. However, we find a strong trend of exon gain over time (Figure 2D; Table 1), supporting the *de novo* model.

Average exon length, on the other hand, shows no clear age-dependence (Figure 2E). Only the youngest genes (ps20) have significantly longer exons (Figure 2E) suggesting a fast secondary acquisition of introns after gene emergence, or gene fusion effects [38].

**Figure 3:**
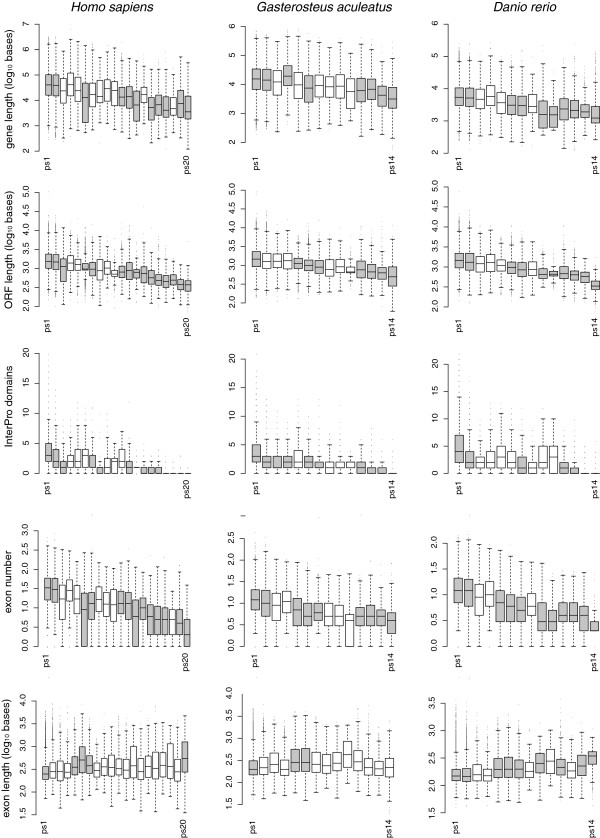
**Trend comparisons in additional genomes.** Same analysis as shown in Figure 2, but for humans (*Homo sapiens*), stickleback (*Gasterosteus aculeatus*) and zebrafish (*Danio rerio*). Note that the fish phylostratigraphy has only 14 phylostrata in total so far, whereby ps1-12 are shared with the mammal genomes. Statistical annotations as in Figure 2.

To assess whether these patterns constitute general trends that can be observed in other lineages as well, we have also analyzed them for humans, stickleback and zebrafish lineages. Humans were included since the genome is equally well annotated as the mouse genome, the fish species represent another vertebrate lineage split more than 400 million years ago. Analysis of these three genomes confirms indeed almost all trends with similarly high correlation coefficients (Figure 3; Table 1). Gene length, ORF length, domain numbers and exon numbers show all a clear time-dependence. Only one comparison, namely the significantly longer exons in the youngest genes was not confirmed for the two fish genomes. However, for these genomes this may in part be due to a bias against annotating genes that have no homologs in other genomes. Note that the shared trends can only partly be ascribed to the shared early history of vertebrates. The fish versus mammal lineages have had 800 million years of independent evolution, during which the trends seen in the genes shared between the lineages could have been subject to changes, unless they were robust.

### Chromosomal distribution

**Figure 4:**
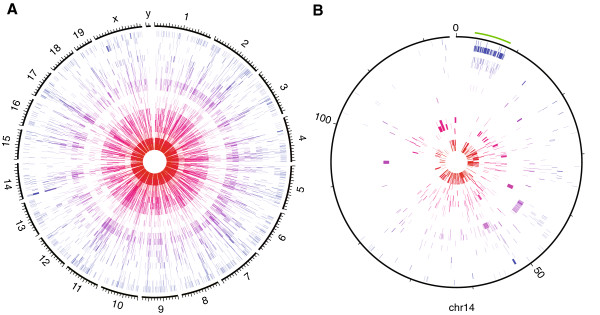
**Circos plots of chromosomes and phylogenetic age of their genes.** Clockwise orientation, tick marks every 10 Mb. Genes become younger towards the outer part of the circle (represented by hues of red to blue). **A**. Whole genome representation. **B**. Chromosome 14. Green mark indicates a local cluster of young genes spanning several phylostrata.

Gene emergence appears to be randomly scattered across all chromosomes (Kolmogorov-Smirnov test, 10,000 permutations), with exception of a few clusters (Figure 4A). However, most of these represent a single locally expanded gene family, with one interesting exception on chromosome 14. This is a block of about 5 Mb located at the centromeric end of the chromosome (Figure 4B). This cluster has already been described as a complex region including a gene family involved in regulating synaptic activity in mouse [39]. Our analysis suggests that it is indeed a region with a high rate of gene birth, composed of sets of genes that have arisen at different times. But, apart of this special region, there is currently no indication for a localized generation of new genes. Hence, although the *de novo* and the duplication-divergence model are both compatible with this pattern, one could have expected for a duplication model that more local clusters could have become apparent.

### Association with transcriptionally active sites

**Figure 5:**
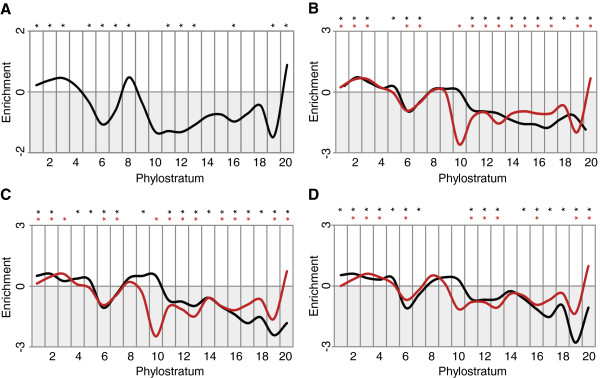
**Association of transcription marks by phylostratum.** Log-odds of gene counts as enrichment. **A**) combination of three transcriptional hallmarks: CpG islands, H3K4me3 peaks and DNAseI sensitivity hotspots. **B**) to **D**) profiles for single transcription marks, separately for unidirectional promotors (black lines) and bidirectional promotors (red lines). **B**) CpG islands, **C**) H3K4me3 peaks, **D**) DNAseI sensitivity hotspots. * Hypergeometric test, FDR corrected, p<0.01.

Transcriptionally active regions can be identified by specific marks, such as CpG islands, histone methylation (H3K4me3) peaks or DNAseI sensitivity hotspots. We find that genes in ps1-3 (representing origin of cellular organisms, eukaryotes and opisthokonts, respectively) have a significant excess of genes associated with these regions (Figure 5A), in line with their predominantly general cellular functions. Another over-representation peak occurs at ps8 (evolution of chordates), which is of yet unclear significance.

With respect to the *de novo* model, it is particularly interesting to ask whether the most recently evolved genes are associated with such marks, since this could imply that they tend to make use of existing promotors upon their emergence. We find indeed a significant over-representation of transcriptional marks for genes that have emerged in ps20 (Figure 5A). This would suggest that the transcription of *de novo* evolved genes is initially often dependent on the proximity to an existing transcriptionally active region. Intriguingly, however, the ps19 genes show a significant under-representation with respect to the association of these three marks. This would suggest that new genes acquire rather quickly own regulatory elements, independent of the standard marks.

To explore this pattern further, we analyzed each of the three marks separately and further distinguished between unidirectional and bidirectional promotors (Figure 5B-C). The latter are the most evident candidates of cases where newly evolved genes take advantage of an existing regulatory region. We find that bidirectional promotors are indeed consistently over-represented in genes from ps20 for all three marks.

### Testis expressed genes

Testis is known to have the largest number of tissue-specifically expressed genes, many of which are newly evolved genes [5]. It has therefore been suggested that new genes arise predominantly first in the context of testis expression, before acquiring roles in other tissues - the “out of testis hypothesis” [5].

**Figure 6:**
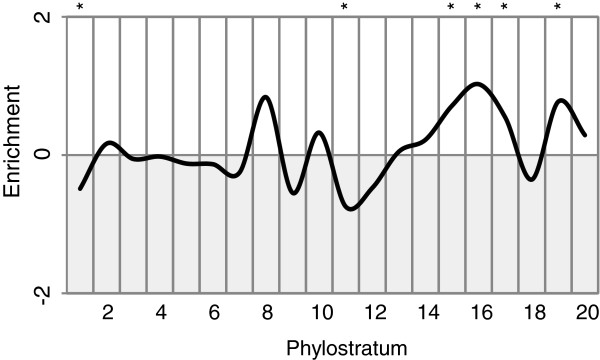
**Phylogenetic profile of genes expressed in mouse testes.** Log-odds of expressed genes as enrichment for each phylostratum. * Hypergeometric test, Bonferroni corrected, p<0.01.

When plotting the over- and under-representation profiles specifically for testis expressed genes, we find a significant enrichment for testis genes mostly from ps15 onwards (Figure 6). But there is no significant peak at ps20 as one would have expected under the “out of testis” hypothesis. On the other hand, it should be noted that we are looking here at protein-coding genes only, while many newly emerged testis expressed genes may initially have been non-coding and have evolved a functional ORF only later on [3]. This hypothesis is in line with the peak seen in ps19, which represents the time frame within which functional ORFs could have evolved.

### Alternative reading frames

**Figure 7:**
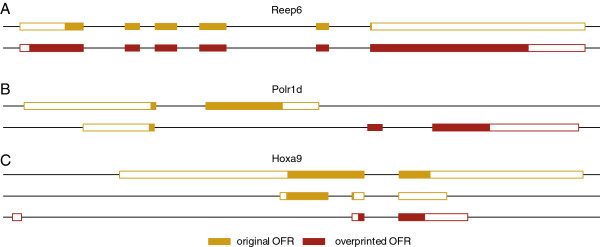
**Examples for overprinting of genes.** Gene structures of four genes are shown, whereby the exons (boxes) are drawn to scale, while the introns (lines) are not to scale. Open boxes are non-coding, filled boxes represent the reading frames. Ancestral gene versions are yellow, derived ones are purple. The Figure is based on annotations and graphics from Ensembl, whereby only the relevant splice variants are shown. **A**: Reep6 (ENSMUST00000030237 and ENSMUST00000060501); **B**: Polr1d (ENSMUST00000154641 and ENSMUST00000114425); **C**: Hoxa9 (ENSMUST00000048680, ENSMUST00000110557 and ENSMUST00000050970).

*De novo* evolution of genes could also occur within the context of an existing gene, for example through the emergence of an alternative exon that changes the reading frame or by making use of a different start codon which would lead to the translation of an alternative reading frame [26,27]. We used the phylostratigraphy approach to assess the age of the ORFs of genes with two annotated reading frames and find that they can indeed be significantly different, indicating a secondary evolution of a new gene within an existing gene. We can find 13 such genes among the current Ensembl annotated reading frames, only two of which were previously identified as overprinted genes (Table 2). We discuss here three further examples representing three general patterns (Figure 7).

**Table 2:** **Spearman’s ρ rank correlation coefficients across phylostrata calculated for the means of the respective distributions (compare Figures**2**and**3**)**

	**Mouse**	**Human**	**Stickleback**	**Zebrafish**
gene length	- 0.88	- 0.90	- 0.82	- 0.93
ORF length	- 0.98	- 0.96	- 0.98	- 0.97
domain number	- 0.94	- 0.91	- 0.72	- 0.90
exon number	- 0.93	- 0.96	- 0.94	- 0.94

All are significant at p < 0.01.

**Figure 8:**
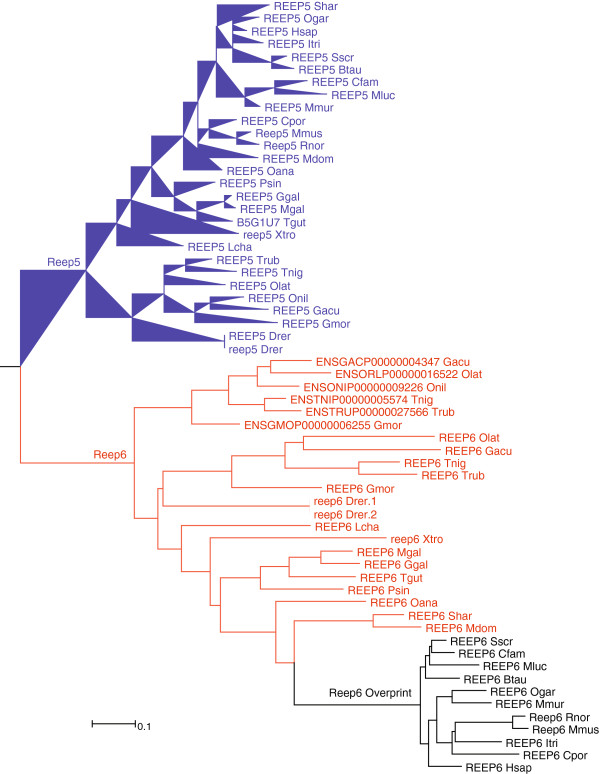
**Phylogenetic tree for Reep5 (blue labels) and Reep6 (red/black labels).** Both genes are present in euteleostomes (ps12), and belong to a larger family of eukaryotic genes (ps2). The Reep6 locus in mouse codes for two proteins of different age. The older protein (Reep6) was mapped to ps2 (eukaryotes), the newer protein (Reep6 overprint) to ps17 (boreoeutherians). Note the enhanced substitution rate at the basis of this subtree (in black), as represented by branch length. Modified gene tree from Ensembl record ENSGT00550000074535.

The first example is the gene Reep6, where an additional start codon has evolved in the first exon, which initiates a new reading frame, overlapping the ancestral one (Figure 7A). The older product of Reep6 maps to ps2, the newer one to ps17, i.e. it appears to have acquired a new function at the boreoeutherian divergence. Interestingly, when looking at the gene trees of these proteins, one can see a clear acceleration of divergence rates in conjunction with the emergence of the second reading frame for Reep6, but not for its nearest paralog Reep5, which has not developed the second reading frame (Figure 8). Such acceleration is a hallmark of an adaptive phase and was also found in viruses [29].

The second example for overprinting is Polr1d, a subunit of RNA polymerase I and III, which has acquired two additional exons at the end of the ancestral gene. Alternative splicing leads thus to a new protein that shares only the start codon and a few initial amino acids with the ancestral gene (Figure 7B). The ancestral protein maps to ps2, the derived one to ps5, i.e. this arrangement with two protein products from the same gene region is highly conserved.

The third example is Hoxa9, one of the canonical Hox genes involved in anterior-posterior patterning. In this case, the ancestral gene has first acquired an additional intron that leads to a truncated version of a protein, an arrangement that is conserved between birds to mammals [40] (ps14). On top of this, an additional 5′-exon, driven by a new promotor, has evolved within the Euarchontoglires (ps18). This splices to the acceptor of the new intron and creates thus a new reading frame (Figure 7C). Interestingly, this reading frame covers the homeobox and is conserved between primates and rodents.

## Discussion

The trends described above provide new insights into the modes of gene emergence over time. For the two models, *de novo* evolution versus duplication-divergence, it seems that *de novo* evolution is better compatible with these trends. But before coming to the interpretations, we should first like to discuss the technical aspects of our approach.

We rely generally on blastp searches for classifying the genes to phylostrata. There have been extensive simulation efforts that have shown that this is an adequate procedure [34]. However, if one would add manual curation, including the use of a combination of different search algorithms, one would indeed classify a number of genes to older phylostrata. On the other hand, we are focusing here on general trends, not on absolute numbers. Given that most of these trends are robust, both with respect to statistical testing, as well as for confirming them for the much less well annotated fish genomes, we consider the possible misclassification problem as small.

We relate our analysis only to the currently annotated Ensembl reading frames, although these are in a constant flux, due to curation and further refinement of annotation procedures. In fact, it has already been noted that the currently available annotations underestimate the number of orphan genes, since finding a homologue for a gene is one accessory criterion for annotation. This affects mostly the genes from ps20, which are under-represented [3,9], although they are the best candidates for ongoing *de novo* evolution. Hence, although some noise is expected in the data and the assignment fidelity, it would be very unlikely that a systematic artifact causes the trends observed.

### De novo evolution versus duplication-divergence

The *de novo* emergence of a gene out of non-coding DNA requires only some form of transcription, as well as simple signals that define its start and its end and possibly splice sites, as well as some open reading frame [3,7]. Since all of these signals are rather short, they are expected to occur frequently even in random sequences. Genes emerging from such random combination of signals have been called proto-genes [7,20] and analysis of ribosome association profiles in yeast has suggested that they are abundantly translated [19,20]. Accordingly, they could easily serve as a continuous source of short genes that are ready to become recruited to functional pathways and can then become more complex over time. Hence, new genes that arise according to this model would initially be short, have few introns and domains and would often be associated with existing regulatory elements. These are indeed the overall trends that we observe.

The duplication-divergence model, on the other hand, seems much less compatible with these trends. Under this model, one would expect that the new gene should inherit the gene structure from the parental gene. Since long and short genes should equally often be the source of new genes, and since duplications should happen similarly at all time horizons, one would not expect to see the dependence between age and length features.

Domain number is also highly correlated with age, with younger genes having far fewer domains. This is not a simple effect of the similarity searches that we have used, since the domain annotation in Interpro is based on a combination of a variety of different procedures that go beyond blastp matches [41]. Hence, this observation confirms that not only new genes, but also new domains can arise over time [42,43]. On the other hand, only half of the genes contain known domains [37], i.e. having a domain is not a prerequisite of protein function. In fact, many proteins are known to be intrinsically unstructured [44-46].

### Regulatory evolution

It is still unclear how a new gene can acquire its regulatory elements. One possibility is that there are many cryptic transcriptional initiation sites around the genome. Indeed, it appears that most of the genome becomes transcribed at some time [47,48]. However, much of this may be co-transcription or spurious initiation. Moreover, to allow a transcript to become functional (i.e. to become subject to positive selection), it requires some form of stable and heritable regulation. We have therefore evaluated the possibility that new genes make use of existing promotors. It is known that RNA polymerase II promotors have a general tendency for divergent transcription within the nucleosome-free region associated with most promotors [49,50]. We find indeed an enrichment of general signatures of active promotors in association with the most recently evolved genes (ps20). This is mostly due to bidirectional promotors, where the general tendency of RNA PolII for bidirectional transcription may have become extended to form a new transcript. Intriguingly, the next phylostratum (ps19) shows an under-representation of genes among bidirectional promotors, which would suggest that a new gene that has become functional could rather quickly gain its own independent promotor elements.

### Overprinting

Another way of making use of an existing promotor is to develop an alternative reading frame within an existing gene. This can be caused by the acquisition of an alternative splicing, whereby the original start codon is retained (e.g. in Polr1d). Alternatively, a separate start codon becomes used that initiates a different reading frame (e.g. Reep6). This has long been thought to be very unlikely, mostly because of the common notion that in eukaryotes only the first AUG serves as a start codon in a mRNA. However, polycistronic mRNAs are known to occur in eukaryotes as well [51], i.e. the use of additional start codons from the same transcript is not without precedence. The third possibility to initiate an alternative reading frame within an existing gene is a new upstream exon, driven by a new promotor, combined with alternative splicing. This has apparently happened in the case of the Hoxa9 gene. This is also the mechanism that was found for the previously well-studied example of overprinting in the Cdkn2a gene [32]. This raises of course the question of how the new promotor for the new upstream exon has evolved. However, it has been shown that there is a widespread presence of long-range regulatory activities in the mouse genome, which can act on inserted promotors [52]. Thus, it seems indeed rather conceivable that random mutations in such potentially active regions might suffice to create a new regulated initiation site.

We expect that it should be possible to detect many more cases of overprinting, if one does not only search annotated reading frames, as we have done here. For example, Chung et al. [33] have identified 40 candidates for overprinting in humans using a probabilistic search strategy. With the much better genome sampling that we have nowadays, it should be possible to refine the searches even further.

Our search has specifically focused on cases where the overprinted reading frame has emerged later than the original one. Two of the previously well-studied genes fall into this class and we have recovered them. Such secondarily evolved proteins are the ones that give the strongest support for a *de novo* evolution mechanism, since alternative reading frames of long existing genes can be considered as almost random sequences. Hence, the fact that new proteins can arise out of them is a strong argument for the reality of *de novo* evolution [26,27,33].

## Conclusion

The phylostratigraphy-based analysis of trends associated with gene emergence in the mouse genome is well compatible with a frequent *de novo* emergence of orphan genes. This seems to be in contrast to previous assessments, which found only a small fraction of cases of *de novo* evolution [10,53,54]. However, it is necessary to emphasize that this depends very much on the criteria that were used. These early studies were still constrained by the assumption that *de novo* evolution must be rare and the criteria were therefore tuned to be very restrictive to be sure that only the best-supported cases were included. In addition, it has initially been unclear whether any new gene that includes part of a transposable element should be classified in a separate class [53], since strictly speaking it contains at least partly a duplicated sequence. On the other hand, if the transposable element fragment does not contribute its reading frame to the new gene, we would now consider it as a *de novo* gene, given that we find also overprinting in other existing genes. We should also reiterate that our analysis here is strictly based on genes that were annotated as protein coding, whereby the criteria for annotation of genes are still rather restrictive and tend not to consider short open reading frames, although these may be functional as well [51]. Further, all non-coding RNAs are still excluded from this analysis, although the emergence of new *de novo* genes may be characterized by a phase where it acts as non-coding RNA first [11,13]. Hence, we conclude that we are only at the beginning to understand the true impact of *de novo* gene evolution on shaping the genome and emergence of new gene functions.

## Methods

### Phylostratigraphy

The phylostratigraphic procedure [21] is a blastp-based sorting of all protein sequences of an organism according to their phylogenetic emergence. The procedure uses the annotated genes of the focal organism and compares them to all available annotated and non-annotated genome data to infer the first time of emergence of a given gene. Accordingly, all available proteins from protein coding loci in the version 66 of Ensembl [55] for *Mus musculus* (obtained through BioMart [56]) were queried against the *nr* database from NCBI using an e-value threshold of < 10^-3^, which has been shown to be optimal for such an analysis [1,34]. For phylostratum 12, given the low number of protein sequences for outgroups (Cyclostomata/Chondrichthyes), EST and Trace data were included in a tblastn query (translated nucleotide comparison), using an e-value threshold of <10^-15^. The computation of the phylostratigraphic maps was performed on the Phylostrat server of the IRB Institute, Zagreb, Croatia. Twenty phylogenetic age classes, i.e. phylostrata, were defined based on consensus phylogenetic relationships (Figure 1). The age of a locus was assigned taking into account the oldest detectable similarity of any of its protein products. This approach is targeted to the detection of orphan genes, as it neglects events of exon shuffling or gene fusion as genomic novelties.

### Gene structure analyses

Structural gene features were obtained from version 66 of Ensembl through BioMart for mouse (*Mus musculus*), and from version 68 for human (*Homo sapiens*), zebrafish (*Danio rerio*) and stickleback (*Gasterosteus aculeatus*). Domain information from Interpro [41] was also obtained through BioMart, and the number of different entries per gene was used as a proxy to the number of domains. Phylostratigraphic analyses were tested with hypergeometric statistics for discrete features and correlations were calculated for continuous features. A combination of permutations (n=10,000) and Kolmogorov-Smirnov tests was used to assess the significance of each phylostratum per variable. Kolmogorov-Smirnov tests were also applied to distance distributions. Other statistical tests were perfomed using R version 2.15.1 [57] and PASW version 18.0.0 [58]. Circular plots for the mouse genome were done with Circos [59].

### Transcription associated regions

Regions of high transcriptional activity from basal promotors were defined as those containing any of these three features: presence of CpG islands, H3K4me3 peaks or DNAseI sensitivity hotspots. These features allow broad range recognition of potential and actual sites with enhanced transcriptional activity. All datasets were taken from the UCSC Genome Browser [60,61] through the Table Browser tool [62]. Datasets for H3K4me3 ChIP-seq (Mouse ENCODE Consortium, 2012) were obtained from the available tracks from *Histone Modification by ChIp-seq* at ENCODE/LICR (Ludwig Institute for Cancer Research). Available tissue data at the time of the study include bone marrow, cortex, cerebellum, heart, kidney, liver, lung, mouse embryonic fibroblasts and spleen (all from 8 week old mice). Only peak data were used. Datasets for DNAseI sensitivity assays were obtained from the *DNAseI Hypersensitivity by Digital DNAseI* from ENCODE/University of Washington tracks [63]. Only hotspots information was used and only tracks corresponding to C57BL/6 mice. Genes were considered to be associated to these marks if the transcription start site was found at a distance of 1,250 bases or less from the mark, accounting for potential offsets in annotations and allowing the assumption that transcriptional activity might affect more drastically those regions in a short range. Analyses of overlap between regions were performed with the BEDtools suite [64]. Phylostratigraphic enrichment was calculated as log-odds and tested using hypergeometric statistics and FDR correction.

### Expression data for testis

Mouse microarray expression data from [65] were obtained from the authors’ website (http://hugheslab.ccbr.utoronto.ca/supplementary-data/Zhang/). This study was selected because of the wide spectrum of tissues considered, which allow for an unbiased measure of expression for a large set of genes. Given that the study was performed using a draft of the mouse genome, the probes were re-annotated using Blat [66] to match the phylostratigraphic map of the mouse. Ambiguous and poorly matching probes were discarded from the analyses.

### Secondary reading frames

This screen was devised to find annotated candidates for emergence of new genes within existing genes based on annotated products. All complete open reading frames corresponding to the same genomic location (ENSMUSG) were considered as candidates, if the minimum and maximum age values differed by at least 2 phylostrata (to avoid screening borderline classifications between phylostrata). Within each genomic location, ORFs were aligned at the nucleotide and protein level using global (needle) [67] and local alignments (blastn and blastp, database size adjusted to emulate nr-sized searches) [68]. The oldest product was used as reference, and any products with younger phylostrata values were used as query. In the case of multiple older products, comparisons were made against all possible products from the oldest phylostratum. Non-matching protein alignments coming from matching nucleotide alignments were considered as genes with alternative reading frames. These were screened manually in Geneious (version 5.6.5) to identify conservation patterns of start and stop codons in other species. Additionally, using the Compara platform from Ensembl [69], phylogenetic trees for selected candidates were analyzed.

## Competing interests

The authors declare that they have no competing interest.

## Authors’ contributions

RN retrieved the data and performed the analyses. RN and DT conceived the study, interpreted the data and wrote the manuscript. All authors read and approved the final manuscript.

## Supplementary Material

Additional file 1: Table S1 Excel workbook with compilation of all loci from mouse, humans, zebrafish and stickleback assigned to their respective phylostrata. Click here for file
